# Current Perspectives of Neuroendocrine Regulation in Liver Fibrosis

**DOI:** 10.3390/cells11233783

**Published:** 2022-11-26

**Authors:** Bowen Li, Hui Wang, Yudian Zhang, Ying Liu, Tiejun Zhou, Bingru Zhou, Ying Zhang, Rong Chen, Juan Xing, Longfei He, Jennifer Mata Salinas, Sachiko Koyama, Fanyin Meng, Ying Wan

**Affiliations:** 1 School of Basic Medical Science, Southwest Medical University, Luzhou 646000, China; 2 Department of Pathology, The Affiliated Hospital of Southwest Medical University, Luzhou 646000, China; 3 Division of Gastroenterology and Hepatology, Department of Medicine, Indiana University School of Medicine, Indianapolis, IN 46202, USA; 4 Richard L. Roudebush VA Medical Center, Indianapolis, IN 46202, USA

**Keywords:** neuroendocrine hormones, neuroendocrine regulation, hepatic stellate cells, tissue homeostasis, liver fibrosis

## Abstract

Liver fibrosis is a complicated process that involves different cell types and pathological factors. The excessive accumulation of extracellular matrix (ECM) and the formation of fibrotic scar disrupt the tissue homeostasis of the liver, eventually leading to cirrhosis and even liver failure. Myofibroblasts derived from hepatic stellate cells (HSCs) contribute to the development of liver fibrosis by producing ECM in the area of injuries. It has been reported that the secretion of the neuroendocrine hormone in chronic liver injury is different from a healthy liver. Activated HSCs and cholangiocytes express specific receptors in response to these neuropeptides released from the neuroendocrine system and other neuroendocrine cells. Neuroendocrine hormones and their receptors form a complicated network that regulates hepatic inflammation, which controls the progression of liver fibrosis. This review summarizes neuroendocrine regulation in liver fibrosis from three aspects. The first part describes the mechanisms of liver fibrosis. The second part presents the neuroendocrine sources and neuroendocrine compartments in the liver. The third section discusses the effects of various neuroendocrine factors, such as substance P (SP), melatonin, as well as α-calcitonin gene-related peptide (α-CGRP), on liver fibrosis and the potential therapeutic interventions for liver fibrosis.

## 1. Introduction

The liver is the largest gland in the body that plays a critical role in maintaining homeostasis of the body and has many important physiologic functions. Hepatocytes occupy 60–80% of the total cell populations in the liver, and the other cells include hepatic stellate cells (HSCs), cholangiocytes, Kupffer cells, liver sinusoidal endothelial cells, hepatic stem/progenitor cells, etc. [1].

Liver fibrosis is the process of abnormal accumulation of extracellular matrix (ECM) and eventual formation of fibrous scar in response to chronic liver injury caused by various diseases, such as excessive alcohol drinking, viral hepatitis, non-alcoholic fatty liver disease as well as cholestasis. Without proper treatment, the fibrous scar formed due to long-term liver fibrosis may destroy the normal structure of the liver and lead to the loss of hepatocytes and dysfunction of the liver, eventually resulting in liver failure or cancer, which seriously threatens human life [2].

Nowadays, the function of neuroendocrine regulation in liver fibrosis is a new territory that provides new clues for the treatment of chronic liver diseases. In previous studies, it has been reported that the liver is innervated by sympathetic, parasympathetic, and peptidergic nerves, including afferent and efferent fibers. Many neuropeptides produced in the liver have been identified [3]; for example, neuropeptide Y (NPY), substance P (SP), and α-calcitonin gene-related peptide (α-CGRP) are abundant in the connective tissue within the lobules [4]. Aberrant expressions of some neuropeptides in the hepatic environment may regulate pathological inflammation and tissue homeostasis, thus promoting or inhibiting the development of liver fibrosis. The liver is also affected by these neural signals as a receptor and effecter. Therefore, clarification of the role of these signaling pathways in the liver is significant for the therapy of liver fibrosis. However, further studies are needed to be performed to elucidate the underlying mechanisms of those neuropeptides’ effect on liver diseases before they can be used as the target to improve hepatic fibrosis. In this review, we focus on neuropeptide production during chronic liver disease and discuss the research advances related to the neuroendocrine regulation involved in liver fibrosis.

## 2. Pathophysiology of Liver Fibrosis Development

In numerous organs of the body, myofibroblasts are indispensable cellular factors in damage repair, but abnormal myofibroblast activation is a major pathogenic factor of fibrosis disease [5]. In liver fibrosis, activation and proliferation of myofibroblasts play an essential role because activated myofibroblasts are the major source of the extracellular matrix (ECM) [6]. Myofibroblasts accumulate at the site of injury and produce ECM. Hepatic fibrosis is the process of abnormal accumulation of ECM in the liver. If there is prolonged liver injury, hepatocytes will be replaced by abundant ECM. It has been reported that activation of HSCs is central to liver fibrosis, as activated HSCs (a-HSCs) are the main, but not the only, source of myofibroblasts.

**Figure 1:**
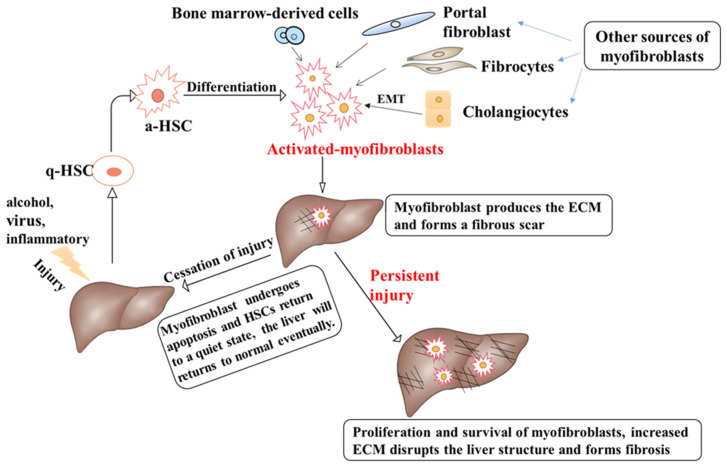
The pathogenesis of liver fibrosis. Physiologically, HSCs are in a quiescent state. Different types of chronic liver injury may activate HSCs and convert them into myofibroblasts. The main source of myofibroblasts is HSCs, and other sources include bone marrow-derived cells, portal fibroblasts, and fibrocytes. Cholangiocytes can be converted into myofibroblasts through EMT. Activated myofibroblasts can produce ECM at the site of injury and form a fibrous scar. If the injury resolves, myofibroblasts undergo apoptosis and senescence, HSCs return to a quiescent state, ECM and scar tissue are degraded, and the damaged liver is repaired. If the injury persists, normal injury repair is disrupted, injury factors will further activate HSCs and myofibroblasts, and ECM will accumulate excessively in the liver, eventually resulting in liver fibrosis.

In normal physiological conditions, fibroblasts are derived from mesenchymal cells [7]. However, there are four different sources of myofibroblasts when there is liver injury [8]. HSCs have been shown to transform into myofibroblasts in liver fibrosis due to various injuries (Figure 1). Other types of cells which become myofibroblasts include bone marrow-derived mesenchymal cells, fibroblasts, as well as portal fibroblasts [9,10] (Figure 1). The activation of myofibroblasts by different cell types depends on the pathogenic factors of liver fibrosis. In carbon tetrachloride (CCL4)-induced liver injury, myofibroblasts (>87%) were mainly derived from HSCs, while activated portal fibroblasts were the main source of myofibroblasts in cholestatic liver injury [11]. Recent studies have also shown that cholangiocytes and hepatocytes may undergo epithelial-mesenchymal transition (EMT) and become myofibroblasts during the development of liver fibrosis [12] (Figure 1). However, other studies demonstrated that no effects of hepatocytes and cholangiocytes EMT were involved in BDL or CCl4-induced liver fibrosis [13,14]. Therefore, the controversy can be rooted in different animal models and treatments of hepatic fibrosis.

The ECM consists of five substances, namely collagen, non-collagenous proteins, elastin, proteoglycans, and aminoglycans. The degree of liver fibrosis is associated with changes in the number and composition of ECM [15]. In patients with advanced hepatic fibrosis, the amount of ECM is six times higher than that in normal liver tissue. The balance between ECM production and degradation is a key factor that determines wound healing and tissue recovery [16], and in the healthy liver, the ECM is precisely regulated by matrix metalloproteinases (MMPs) and their specific inhibitors, tissue inhibitors of metalloproteinases (TIMPs), to maintain homeostasis. Activation of myofibroblasts upregulated the expression of TIMPs and inhibited the activity of MMPs, leading to the accumulation of ECM [17]. The accumulation of ECM is due to increased synthesis and decreased degradation of the ECM [18], eventually leading to the development of liver fibrosis.

Under physiologic conditions, HSCs remain quiescent and are responsible for the storage of vitamin A in the liver. Upon various types of chronic injury caused by alcohol, virus, nonalcoholic steatohepatitis, and other factors, HSCs may be activated, and transit into myofibroblasts, and the latter secrete abundant extracellular proteins [19]. Thus, they acquire contractile, pro-inflammatory, and fibrotic properties that contribute to the development of liver fibrosis [20]. The altered phenotype of HSCs following injury stimuli is generated at the level of gene expression in response to liver injury, and the activation of HSCs is driven by paracrine stimuli and products released from the damaged hepatocyte. Long-term injury stimulation results in sustained activation of the HSCs [21]. All these behaviors contribute to ECM accumulation during the progression of hepatic fibrosis.

Cholangiocytes are the epithelial cells arranged in the bile ducts and are the main target cells for bile duct diseases, which are characterized by the cholangiocyte proliferation [22,23]. Cholangiocyte proliferation is closely associated with ECM production. There are many cytokines and growth factors released through proliferating cholangiocytes that regulate the activation, proliferation, and migration of HSCs and increase the accumulation of ECM.

It should be noted that liver fibrosis may be improved because HSCs may undergo cellular senescence, apoptosis, or return to a quiescent state when injury stimuli are alleviated or removed [24]. Degradation of the ECM can occur by decreasing the activity of TIMPs and increasing the MMPs activity [5]. The loss of myofibroblasts is not the only component of liver fibrosis reversal; macrophages also play an important role in the progression of liver fibrosis. Macrophages induce the conversion of HSCs to myofibroblasts and promote liver fibrosis. While in the decline of liver fibrosis, macrophages decompose ECM by producing MMPs [25].

## 3. Hepatic Innervation and Neuroendocrine Compartments Are Involved in the Chronic Liver Diseases

The unique nervous system of the liver during liver fibrosis has been extensively studied. The liver contains both efferent and afferent nerves that can be affected by catecholamines, acetylcholine as well as neuropeptides. The efferent nerve originates in the hypothalamic nucleus and controls the transmission of various factors into the liver. Afferent sensory nerves transmit cytokine and metabolite information via the vagal pathway or transmit pain information to the central nervous system via the lower thoracic pathway. They are responsible for transmitting signals in the brain/hepatic nerve axis. Thus, the liver acts as a sensor and effector and is influenced by these neural signals [3].

Neuroendocrine cells are a type of nerve cells that can secrete hormones in the organism. They are structurally part of the nervous system rather than the endocrine system but display endocrine cell-like activity. They can secrete neurohormones and regulate the functions of other organs through blood circulation or local diffusion. Neuroendocrine cells are located in multiple organs of the body and exhibit similar structural, functional, and metabolic properties as endocrine phenotypes from neurons. Typically, neuroendocrine cells comprise secretory granules, called large dense-core vesicles, and express protein markers such as chromogranin-A, neural cell adhesion molecules, neuron-specific enolase, and synaptophysin [26]. Chromogranin-A has been reported to be an exceedingly reliable marker for neuroendocrine cells. These markers display diverse specificities as well as sensitivities in recognition of the neuroendocrine phenotype of cells and are involved in different intracellular activities, for example, packaging of hormones and regulation of neuropeptides release [26]. Neuroendocrine cells or tissues can produce a variety of neuropeptides, such as serotonin, endogenous opioid peptides, growth inhibitors, as well as calcitonin, which have many functions, including vasoconstriction, vasodilation, and regulation of cellular proliferation and apoptosis [27].

Studies have demonstrated that local neuroendocrine factors exert an effect on fiber production in many organs of the body [28]. Neuroendocrine regulation in the liver is usually associated with injury stress, and inflammatory factors and ECM components can induce a neuroendocrine phenotype of cholangiocytes [26]. In terms of the ability of the neuroendocrine regulation of cell proliferation and apoptosis, the various neuroendocrine-like cells in chronic liver injury may regulate cell proliferation, apoptosis, migration, and angiogenesis during liver fibrosis.

### 3.1. Cholangiocytes

The majority of bile duct injuries trigger cholangiocyte proliferation in chronic liver injury. Abnormal proliferating cholangiocytes acquire a neuroendocrine-like phenotype to maintain hepatic homeostasis by secreting different cytokines, growth factors, neuropeptides, and hormones [23]. This process in biliary injury triggers cholangiocytes proliferation and is called ductular reaction. The ductular reaction is often present in chronic liver disorders, which are associated with enhanced necroinflammatory activity and fibrosis stage. The proliferation of cholangiocytes is generally prior to that of other cell behaviors of liver diseases. It has been demonstrated that activated cholangiocytes are able to recruit inflammatory cells as well as myofibroblasts to the point of injury and secrete ECM, pro-fibrogenic factors, leading to scar and fibrosis formation [29]. Recently, the neuroendocrine regulation effect of cholangiocytes in liver injury has been extensively studied.

### 3.2. Hepatic Stellate Cells

Hepatic stellate cells are the special mesenchymal cells residing in the space of Disse and interact with liver sinusoidal endothelial cells (LSECs) and hepatocytes [30]. HSCs are central to the process of liver fibrosis, and the proliferation and activation of HSCs can regulate the amount of ECM and promote the development of liver fibrosis. HSCs display neuroendocrine features during the development of fibrosis because they express many of the same protein markers as the neuroendocrine cells secrete during liver fibrosis [31]. They express synaptophysin, glial fibrillary acidic protein, nestin, neural cell adhesion molecule, neurotrophins, as well as their receptors. Synaptophysin is one of the factors related to neuroendocrine differentiation of the HSCs [32]. Neuropeptides produced by HSCs could maintain the activated myofibroblast phenotype. Different liver injury induces a different change of neuropeptides and their receptors in HSCs, which makes them susceptible to neuroendocrine regulation during the progression of liver fibrosis.

### 3.3. Hepatic Progenitor Cells

Hepatic progenitor cells are distributed in the bile ducts and intrahepatic bile ducts of the portal vein. These cells are activated in response to liver injury and have a bidirectional potential to differentiate into hepatocytes as well as cholangiocytes. When hepatocyte proliferation is inhibited, they will differentiate into cholangiocytes [33]. Progenitor cells express chromogranin-A, neural cell adhesion molecules, parathyroid hormone-related peptides, neurotrophic factor, and neurotrophic factor receptors [34]. This neuroendocrine phenotype suggests that hepatic progenitor cells are the neuroendocrine compartment in the liver and may be under the control of the central nervous system during chronic liver injury.

## 4. The Role of Neuroendocrine Regulation during Liver Fibrosis

### 4.1. Renin-Angiotensin System

The renin-angiotensin system (RAS) is considered a hormonal system primarily responsible for blood pressure control and fluid homeostasis in the body [35]. Renin, an enzyme first described in the kidney, is also found in the brain. The sympathetic nervous system is amplified during stressful stimuli and with numerous downstream effects, including on the RAS because sympathetic nerves strictly control renin production. The RAS is important in regulating organ function through autocrine, paracrine, or endocrine actions. The classic RAS axis is the conversion of renin to angiotensin II (Ang II) via an angiotensin-converting enzyme (ACE). Ang II exerts its biological effects by binding to two types of receptors, namely angiotensin type 1 receptor (AT1) and AT2 [36]. Several research studies have improved our understanding of RAS, including the identification of Ang-(1-7), a biologically active member of the RAS [37]. Ang-(1-7) is produced by the degradation of Ang II by ACE2, a homolog of ACE [38]. Ang-(1-7) exerts its biological action by binding to the G-protein-coupled receptor Mas [39]. Many studies have pointed out that RAS plays an important role in the development of liver fibrosis. One study reported that the RAS component was significantly elevated in the circulation of bile duct ligation (BDL) rats, with a three-fold increase in Ang II and a two-fold increase in Ang (1-7) compared to normal rats [40]. The classical ACE-Ang II-AT1 axis plays a pro-fibrotic, pro-inflammatory effect in the process of liver fibrosis; however, the ACE2-Ang-(1-7)-Mas axis has a counter-regulatory effect on Ang II to improve liver fibrosis [41]. Accordingly, the balance between both RAS axes likely influences the progression of liver fibrosis.

In the liver, the RAS components such as renin and ACE are present in the Kupffer cells [42]. Ang II induces contraction of a-HSCs through AT1 receptors and promotes the proliferation of a-HSCs by activating the mitogen-activated protein kinase (MAPK) pathway, and these effects can be blocked by losartan, a specific AT1 antagonist [43]. Activation of AT1 by Ang II also increases the expression of type I collagen genes, which require activation of both the MAPK and transforming growth factor-β (TGF-β) signaling pathways [44]. Studies have reported that the RAS affects the development of liver fibrosis by regulating the balance of the oxidative stress [45]. Ang II increased NADPH oxidase (NOX) protein and reactive oxygen species (ROS) expression in HSCs, leading to the activation of NOD-like receptor protein 3 (NLRP3) inflammasome in HSCs. However, Ang-(1-7) improves BDL-induced liver fibrosis by decreasing the NOX protein and NLRP3 inflammasome levels and increasing the expression of antioxidants, such as glutathione [45]. Ang(1-7) not only counteracts the oxidative stress effects of Ang II but also counter-regulates Ang II-mediated cell contraction, proliferation, and pro-fibrotic effects [41]. In addition, treatment with the Mas receptor antagonist (A-779) aggravated hepatic fibrosis, further confirming the protective effect of Ang-(1-7) [40]. In general, two axes of the RAS are involved in the development of hepatic fibrosis and play completely opposite roles, and the balance between these two axes determines the direction of liver fibrosis development.

### 4.2. Cannabinoid System

The cannabinoid system consists of specific cannabinoid receptors (CB1 and CB2) and exogenous and endogenous (endocannabinoids, ECs) ligands. Studies have reported that CB1 and CB2 receptors are abundantly expressed in neurons as well as central and peripheral immune cells, which are involved in the modulation of inflammatory neurodegenerative diseases [46], and both CB1 and CB2 are weakly expressed in normal liver tissue [47]. The most profoundly studied ECs are anandamide and 2-arachidonoylglycerol, both belonging to the family of endogenous arachidonic acid-derived ligands [46]. When intracellular calcium levels are elevated in postsynaptic neurons, the enzymes that synthesize ECs are activated, leading to the formation and release of ECs. ECs cross the synaptic cleft and stimulate CB1 receptors on the presynaptic membrane to exert their functions [48]. Subsequent studies showed that the expression of ECs was significantly upregulated, probably produced by hepatocytes and nonparenchymal cells in the chronic liver disease [49]. The expression of CB1 and CB2 was higher in transdifferentiated HSCs than in normal livers [50]. These results suggest that the cannabinoid system is involved in the development of liver fibrosis. The latter study on cannabinoid receptors in liver fibrosis found that CB1 and CB2 play differential effects in the process of liver fibrosis [51].

It has been reported that cannabinoid receptors may be activated by ECs. In three mouse models of liver fibrosis (chronic CCl4 intoxication, chronic thioacetamide intoxication, and BDL), CB1 antagonist (SR141716A) and knockout of CB1 gene alleviated liver fibrosis by reducing the expression of fibrosis markers TGF-β and α-smooth muscle actin (α-SMA). However, the determining factor for the improvement of liver fibrosis by CB1 ablation is the decrease in accumulation and increase in apoptosis of myofibroblasts [50]. Another study reported that CB2 ablation aggravated liver fibrosis in chronic CCl4 intoxication mice, suggesting an anti-fibrotic function of CB2. CB2 ameliorates liver fibrosis by inhibiting the growth of hepatic myofibroblasts and promoting apoptosis of hepatic myofibroblasts as well as HSCs. Growth inhibition involves cyclooxygenase-2(COX-2), and oxidative stress leads to apoptosis [51].

In addition, ECs can act independently without dependence on cannabinoid receptors. Anandamide mediates cell necrosis by promoting the formation of ROS, and the increase of intracellular Ca^+^ and pretreatment with antioxidants and Ca^+^-chelators attenuated HSCs death. Furthermore, cannabinoid receptor antagonists did not resist anandamide-mediated necrosis of HSCs, suggesting that anandamide can exert antifibrotic effects alone [52].

### 4.3. Melatonin

Melatonin is a neurohormone synthesized in the pineal gland by *N*-acetyltransferase (AANAT) and is participated in the regulation of many diverse physiological functions, including the promotion of sleep, circadian rhythms as well as neuroendocrine processes. Melatonin has also been detected in a variety of organs, such as the brain, retina, gastrointestinal tract, and liver [53]. In addition, melatonin has powerful antioxidant and anti-inflammatory functions and modulates mitochondrial homeostasis, which has led to a growing number of studies on melatonin. Several studies suggest that melatonin may play an important role in the treatment of liver fibrosis. Melatonin in the liver may partly come from the gastrointestinal tract via the hepatic portal vein and also from the hepatocyte nuclei [53]. The effects of melatonin are mediated by two signaling mechanisms, including receptor-mediated and non-receptor-mediated pathways.

Cholangiocytes have been reported to express melatonin receptors (MT1 and MT2) [54]. It was reported that melatonin interacted with MT1 in BDL rats to reduce intrahepatic bile duct mass (IBDM) caused by cholangiocytes proliferation via down-regulation of the cyclic adenosine 3′,5′-monophosphate (cAMP) signaling pathway, and also reduced serum transaminase levels, however, these results could only be blocked by MT1 antagonists but not MT2 antagonists, suggesting that the antifibrotic effects of melatonin are mediated by MT1 [54]. Another study reported that intraperitoneal administration of melatonin to CCl_4_-treated mice counteracted a range of CCl_4_-mediated pro-fibrotic factors, such as upregulation of the TGF-β/Smad signaling pathway and increased expression of collagen I and III genes and activation of HSCs. Moreover, melatonin significantly increased the expression of nuclear factor erythroid2-related factor 2(Nrf2) protein, an important regulator of cellular antioxidant response [55]. Next, the mechanism of melatonin suppresses of HSCs has been investigated. It was found that the nuclear melatonin sensor retinoic acid receptor-related orphan receptor-alpha (RORα) was expressed in HSCs, and melatonin directly suppressed HSC activation via RORα-mediated inhibition of 5-lipoxygenase expression, while the RORα antagonist SR1001 blocked the antifibrotic profile of melatonin [56].

In recent years, the effect of mitochondria in liver fibrosis has attracted a great deal of interest. Mitochondrial dysfunction occurs in all types of liver injury. Chronic CCl4 exposure impaired mitophagy and mitochondrial biogenesis and also mediated the reduction of mitochondrial fission and fusion-associated proteins. Melatonin ameliorates CCL4-mediated liver fibrosis by resisting the impairment of mitophagy and mitochondrial biogenesis [57]. In the multidrug resistance gene 2 knockout (Mdr2^−/−^) mice, administration of the AANAT antagonist miR-200b attenuated the expression of AANAT and melatonin, resulting in increased biliary proliferation, angiogenesis, and hepatic fibrosis. In contrast, overexpression of AANAT or inhibition of miR-200b restored the antifibrotic effects of the melatonin [58]. Overall, extensive studies have demonstrated the powerful antifibrotic effects of melatonin, and it is promising for melatonin to be used as an anti-fibrotic drug in clinics.

### 4.4. Substance P

Substance P (SP) consists of 11 amino acids and belongs to the tachykinin family. The tachykinin family has six members, including SP, neurokinin A, neurokinin B, neurokinin K, neurokinin γ, and heme kinin, and SP is encoded by the TAC1 gene [59]. After synthesis, SP is packed in vesicles and transported to the central and peripheral endings of primary sensory neurons. SP can be detected in the central nervous system, sensory nerves, immune cells, and peripheral organs such as the liver and lungs. SP exerts its regulatory effects by binding to tachykinin receptors, which are G protein-coupled receptors of three types: neurokinin-1 receptor (NK-1R), NK-2R as well as NK-3R. However, SP has a high affinity to NK-1R and preferentially binds to it, exerting a series of physiological and pathophysiological regulatory functions, including pro-inflammatory and pro-proliferative effects [59]. Several studies have reported that the SP/NK-1R axis is involved in the fibrotic program of various organs. In this review, we mainly elaborate on the role of the SP/NK-1R axis in liver fibrosis.

It has been identified that SP nerve fibers are abundant in hepatic portal vascular membranes and intralobular connective tissue [4]. It was found that increased expression of TAC1 and NK-1R was measured in the total liver of Mdr2^−/−^ mice and primary sclerosing cholangitis (PSC) patients, and serum levels of SP were also higher than the normal level. Administration of SP to WT mice also increased IBDM and hepatic collagen deposition suggesting the pro-fibrotic effect of SP [60]. However, the SP antagonist (L-733, 060) reduced the serum levels of transaminases and various fibrosis indicators in Mdr2 mice, which ultimately improved the fibrosis [60]. In addition, the knockdown of NK-1R inhibited IBDM, the expression of type I collagen, and α-SMA in BDL-induced cholestasis mice [61]. Wan and her colleagues examined the senescence of cholangiocytes and HSCs and found that the pro-fibrotic function of SP was mediated by increasing the senescence of cholangiocytes and decreasing the senescence of HSCs [60]. Moreover, SP may induce the proliferation and activation of HSCs through the TGF-β1/Smad3 signaling pathway [62].

Interestingly, a recent study reported the anti-fibrotic effect of SP. LSECs account for approximately 3% of the total liver volume and exert anti-inflammatory and anti-fibrotic effects on liver tissue through paracrine action. Nitric oxide (NO) from LSECs is a major factor in maintaining LSEC activity, which reverses a-HSCs to quiet HSCs (q-HSCs) and attenuates liver fibrosis. In addition, hepatocyte growth factor (HGF) secreted by LSECs plays an active role in liver regeneration as a mitogen in hepatocytes. However, the inflammatory environment mediated by liver injury leads to endothelial dysfunction, which reduces the production of NO or HGF. In that study, SP was found to stimulate cell proliferation and NO/HGF secretion in LSECs under both normal and inflammatory conditions, resulting in the inhibition of the liver fibrosis [63]. These contradictory results may be related to the course of the disease and the different SP concentrations used in the experiment or targeting different liver cells, and further studies are needed to elucidate this controversy.

### 4.5. Serotonin

Serotonin, also known as 5-hydroxytryptamine (5-HT), is a biogenic amine that exerts its biological activities by binding to seven major receptor families (5-HT_1_ to 5-HT_7_). 5-HT is best known for its functions in the brain, where it functions as a neurotransmitter at neuronal synapses affecting numerous neurophysiological functions, including learning and memory, pain, appetite, and mood. In addition, the enterochromaffine cells in the gastrointestinal tract are the major site of 5-HT synthesis. 5-HT in the blood is taken up through specialized serotonin transporter (SERT) and stored in the platelets [64]. In response to various stimuli, they accumulated in the damaged tissue, and 5-HT was released. Emerging research suggests that serotonin plays a crucial role in liver diseases. The positive effect of serotonin is demonstrated by platelet-derived serotonin initiating liver regeneration after partial hepatectomy and also promoting tissue repair after ischemia/reperfusion injury [65]. However, serotonin mainly plays a pro-fibrotic role in the development of liver fibrosis.

Expression of 5-HT_1B_, 5-HT_1F_, 5-HT_2A_, 5-HT_2B,_ and 5-HT_7_ receptors was detected in rat and human q-HSCs. However, the expression of 5-HT_2A_ and 5-HT_2B_ was significantly increased in a-HSCs suggesting that the 5-HT_2_ family receptor is closely associated with fibrosis. In addition, a-HSCs were found to express SERT to uptake, release, and respond to 5-HT. The synergistic effect of serotonin and platelet-derived growth factor (PDGF) promotes the activation and proliferation of HSCs. Selective antagonists of the 5-HT_2_ receptor induce apoptosis of a-HSCs and inhibit proliferation of a-HSCs [66]. One study reported that 5-HT depletion attenuates liver fibrosis by reducing the inflammatory response, oxidative stress damage, and downregulating the TGF-β1/Smad signaling pathway [67]. Interestingly, a recent study reported the antifibrotic effect of 5-HT_7_, which is the last identified member of the 5-HT receptor family, in the process of liver fibrosis. 5-HT_7_ expression was downregulated in the liver of CCL4-treated mice. However, the administration of 5-HT_7_ receptor agonist (LP-44) in CCL4-treated mice decreased serum levels of transaminases, oxidative stress injury, and fibrosis markers [68]. In general, 5-HT interacts with different receptors exerting different effects. Therefore, the role of 5-HT on liver fibrosis needs further study.

### 4.6. Calcitonin Gene-Related Peptide

Calcitonin gene-related peptide (CGRP) is a neuropeptide containing 37 amino acids, which are mainly produced in sensory neurons but are also present in various organs, including liver [69] and adipose tissue [70]. Two isoforms of CGRP are named α-CGRP and β-CGRP. The CGRP receptors are located in the brain, heart, lung, spleen, and liver [71], consisting of three subunits: calcitonin receptor-like receptor, receptor activity-modified protein 1, and receptor component protein. In mice and rat liver, α-CGRP-positive innervation forms a dense network in the fibromuscular layer of the biliary tree around the portal vein and in the stromal compartment of portal areas [72]. Furthermore, hepatocytes produce CGRP when there is liver disease such as cirrhosis [73]. α-CGRP levels were found to be higher in the serum of BDL mice than that in control mice, suggesting a close association between CGRP produced by hepatocytes and afferent nerves in the liver and liver fibrosis [74].

It has been reported that α-CGRP induces cholangiocytes proliferation and biliary masses in BDL mice by upregulating cAMP levels, and knockout of α-CGRP resisted these changes [74]. Moreover, α-CGRP promotes the activation of protein kinase C (PKC), leading to the activation of MAPKs and C-Junn-terminal protein kinase (JNK), which is involved in the stimulation of biliary hyperplasia and the development of renal fibrosis [75]. A study reported that α-CGRP depletion alleviated liver injury and fibrosis in BDL mice, which was related to enhanced cellular senescence in HSCs, reduced senescence in cholangiocytes, and reduced activation of the MAPK signaling pathway. In addition, these fibrotic changes were reduced when HSCs were treated with cholangiocytes supernatant from BDL α-CGRP^−/−^ mice, suggesting that α-CGRP may regulate liver fibrosis by autocrine or paracrine means [76]. In general, α-CGRP plays an important role during liver fibrosis, and the elimination of α-CGRP may be an emerging means against liver fibrosis.

### 4.7. Neuropeptide Y

Neuropeptide Y (NPY) is a neurotransmitter that is normally produced in the brain as well as in neurons throughout the gastrointestinal tract and with a high concentration in the biliary tree [77]. NPY was also found in the cytoplasm of pericentral hepatocytes and cholangiocytes in the normal liver. However, more NPY was expressed in bile ducts of the cirrhotic liver [78]. NPY exerts its various functions via six main receptor subtypes (Y1–Y6). The expression of NPY in the hypothalamus can be regulated by ghrelin, which is an endogenous peptide produced in the gastrointestinal tract. Ghrelin can exert orexigenic action by regulating NPY production in the arcuate nucleus [79]. Overexpression of NPY in the hypothalamus can lead to hyperphagia and obesity in rats [80]. However, there is no report about whether ghrelin can influence liver fibrosis progression through regulating NPY expression, although ghrelin has been reported to exert an anti-fibrotic and anti-inflammatory effect on the liver [81].

It has been reported that increased local release of NPY reduced the growth and invasion of cholangiocarcinoma cells [82]. In addition, One study showed that NPY and NPY receptor levels were significantly increased in BDL mice, and Y1–Y5 receptors were detected in cholangiocytes but not Y6 receptors. Furthermore, chronic NPY treatment counteracted the increased transaminase levels, cholangiocytes proliferation, and IBDM in BDL mice [83]. These findings suggest that NPY may improve liver fibrosis by inhibiting the process of cholestasis. Subsequently, a recent study elaborated on the relationship between NPY and HSCs. HSCs synthesize and express NPY and Y1 receptors. In addition, NPY significantly promoted fibrotic responses, such as proliferation and migration of HSCs, via the Y1 receptor, and pre-treatment with Y1 receptor antagonist (BIBP3226) blocked the promotion role of NPY [84]. It is perplexing that the proliferation of cholangiocytes is a key component of cholestatic liver fibrosis. These two conclusions seem to contradict each other. The possible explanation is that NPY binds to different receptors or that the expression of the receptors is distinct at different stages. Overall, the role of NPY during liver fibrosis and its mechanisms need to be further investigated.

### 4.8. Endogenous Opioid Peptides

Opioid peptides are produced in the hypothalamus and other areas of the brain [85] and are also present in peripheral nervous systems, cardiovascular and gastrointestinal systems, and immune cells [86]. Opioid peptides, a neurohormone, have specific receptor-mediated growth-regulating functions in neurons as well as non-neurons cells and tissues. Currently, three members of the opioid peptide family are confirmed: enkephalins, endorphins, as well as dynorphins, each derived from a different precursor peptide [87]. Endogenous opioid peptides exert their hormonal effects through binding to three subtypes of opioid receptors (OR): μ OR, δ OR, and κ OR, all of which belong to the G protein-coupled receptor superfamily.

Several reports illustrate the involvement of opioid peptides in the process of various liver diseases [88]. During cholestasis, cholangiocytes express κOR, δOR, and μOR, and there was a significant increase in opioid peptide levels in both plasma and liver [89], while opioid receptor expression is reduced in the brain, which occurs as an outcome of high ligand levels [90]. Interestingly, the liver can produce Met-enkephalin, especially for cholangiocytes in rats with cholestasis [91] and patients with primary biliary cirrhosis (PBC) [92]. Studies have reported that δOR activation substantially reduces cholestasis-mediated cholangiocytes proliferation and functional responses, and treatment with opioid peptide blockers (Naltrexone) promotes the proliferation of cholangiocytes. Whereas, μ OR activation slightly increases cell growth. The δ OR signal is mediated by the IP3/CamKIIα/PKCα pathway, which inhibits the cAMP/PKA/ERK1/2/AKT cascade [89]. Similar to NPY, opioid peptides exhibit completely different effects on cholangiocytes and HSCs. There is a study that reported that opioid peptides could stimulate the proliferation of HSCs and collagen production. Three opioid peptide receptors are detected in a-HSCs; however, δOR is the only opioid peptide receptor that declines during HSCs activation. Opioid peptides promote HSCs growth and collagen accumulation mediated by activation of the calcium-dependent PKC/ERK/PI3K pathway [93]. Naltrexone can significantly improve the development of liver fibrosis in BDL rats by reducing the activation of HSCs in BDL rats and oxidative damage of cholangiocytes [94]. The effects of opioid peptides on both types of cells may be related to the level of δOR expression since the reduction of cholangiocytes proliferation by opioid peptides is mediated by δOR, which is decreased during the activation of HSCs.

### 4.9. Galanin

Galanin is a 29 amino acid neuropeptide found in small intestine and widely distributed in the amygdala, hypothalamus, locus coeruleus as well as sacral spinal cord. It is involved in many different biological functions [95]. Galanin acts through one of three G-protein-coupled receptors: galanin receptor 1 (GalR1), galanin receptor 2 (GalR2), and galanin receptor 3 (GalR3). GalR1 is distributed in the basal forebrain, hypothalamus, and spinal cord. While GalR2 is more widely distributed in the brain, pituitary, and peripheral tissues. GalR3 has been shown to be expressed only in discrete brain regions [96]. In 2017, Matthew McMillin and his colleagues began researching the relationship between galanin and liver disease. They found that the levels of galanin in serum and liver were significantly elevated in BDL rats. Furthermore, large amounts of galanin were produced by cholangiocytes in BDL rats. Galanin promotes IBDM and cholangiocytes proliferation by interacting with GaR1 expressed in cholangiocytes. In addition, the inhibition of galanin counteracted these changes [97]. Subsequently, they detected the expression of GalR2 in HSCs. Galanin treatment increased HSCs proliferation and fibrogenesis in wild-type and Mdr2^−/−^ mice. Inhibition of Gal1R and GalR2 in Mdr2^−/−^ mice reduced IBDM and the expression of fibrosis markers, whereas GalR2 antagonist (M871) only attenuated liver fibrosis without changing IBDM, suggesting that the promotion of HSCs activation and fibrosis gene expression by galanin is mediated by Gal2R [98].

Overall, restrain of galanin may be a promising treatment for chronic liver diseases. However, another study reported that galanin inhibited the proliferation of a-HSCs to attenuate the development of fibrosis, possibly mediated by GalR2. In this report, galanin treatment significantly inhibited the proliferation of HSC in rats and also suppressed the expression of TGF-β and α-SMA [99]. What could have caused such opposite results requires further research to explain. However, it is demonstrated that galanin plays an important role in the development of liver fibrosis.

### 4.10. Secretin (Sct)

Secretin was originally found in the duodenal S cells. In addition, secretin is also considered to be a neuropeptide hormone because it is also expressed in the brain and regulates the function of the central nervous system [100]. Secretin exerts its function by binding to the secretin receptor (SR), and Sct/SR is expressed only in the large cholangiocytes of the liver [101]. Meng and his team found that the Sct/SR levels in liver sections from PSC patients were significantly higher than in healthy samples. Their study reported that Sct promotes hepatic fibrogenesis in normal and BDL mice, and knockdown of SR or SR antagonist (sec 5–27) treatment ameliorated liver fibrosis. The Sct/SR axis increases the secretion of TGF-β from cholangiocytes, which activates HSCs and promotes fibrosis by paracrine means. Knockdown SR and SR antagonist (sec 5–27) treatment attenuated serum transaminase levels, IBDM, TGF-β, and fibrosis gene expression in BDL and Mdr2^−/−^ mice [102]. In addition, they found that secretin-stimulated TGF-β increased cholangiocytes senescence and decreased HSCs senescence via a paracrine pathway, further promoting liver fibrosis. However, the downregulation of SR counteracted these changes in BDL and Mdr2^−/−^ mice [103]. These experimental data fully illustrate the role of secretin in the progression of liver fibrosis, and targeting the Sct/SR axis may provide a new therapeutic approach for liver fibrosis as well as bile duct disease.

### 4.11. Other Neuroendocrines Involved in Liver Fibrosis

Histamine is a neurotransmitter primarily released from activated mast cells and interacts with histamine receptors (H1–H4) [104]. Histidine is converted to histamine by l-histidine decarboxylase (HDC), an enzyme expressed mainly by cholangiocytes in the liver [104,105]. Once released, histamine is either used or rapidly stored within mast cells. It was found that histamine/histamine receptor levels were elevated in Mdr2^−/−^ mice and PSC patients, and treatment of histamine receptors antagonists (mepyramine or ranitidine) in Mdr2^−/−^ mice reduced biliary tract injury and fibrosis compared to saline treatment [106]. In addition, depletion of HDC improved liver injury, ductular reaction, inflammation, and fibrosis in Mdr2^−/−^ mice [107]. These studies demonstrate that the HA/HDC axis may be a potential target for the treatment of liver fibrosis.

Cortistatin is a cyclic neuropeptide, which is discovered in the brain cortex and hippocampus with anti-inflammatory and anti-fibrotic effect [108]. Cortistatin can alleviate fibrotic responses in various inflammatory disorders by reducing the production of inflammatory factors [109]. Analysis using public databases demonstrated that the expression of cortistatin was obviously lower in the fibrotic liver compared to normal liver tissue [110]. In 2021, a study showed that cortistatin and cortistatin receptors were expressed in HSCs and that cortistatin-deficient mice undergoing hepatotoxic and cholestatic injury treatment showed a higher mortality rate. Cortistatin treatment reversed these exaggerated fibrotic changes and protected against the development of liver fibrosis after liver injury [111]. However, the role of cortistatin in liver fibrosis has not been fully elucidated and the related mechanisms need further investigation.

## 5. Conclusions and Prospects

**Figure 2:**
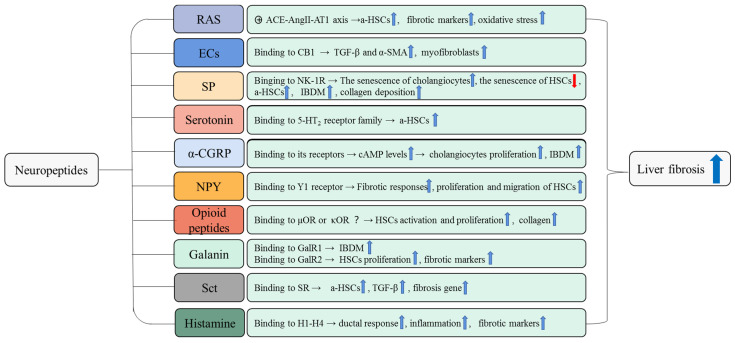
The pro-fibrotic role of neuropeptides in liver fibrosis. The pro-fibrotic effects of neuropeptides and the underlying mechanisms that promote liver fibrosis are summarized in this figure. For example, RAS induces activation and proliferation of HSCs and upregulates the expression of fibrosis markers and oxidative stress damage to promote liver fibrosis. These pro-fibrotic effects of RAS are mediated by the ACE-AngII-AT1 axis. a-HSCs, activated hepatic stellate cells; IBDM, intrahepatic bile duct mass.

**Figure 3:**
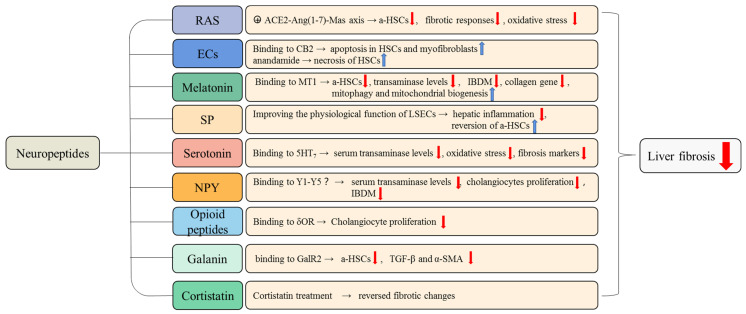
The anti-fibrotic role of neuropeptides in liver fibrosis. The neuropeptides that have anti-fibrotic effects and the underlying mechanisms of these anti-fibrosis effects are summarized in this figure. For example, when binding to the MT1 receptor, melatonin attenuates liver fibrosis by inhibiting the activation of HSCs, reducing transaminase levels, IBDM, and collagen gene expression. The RAS inhibits the activation of HSCs and reverses pro-fibrotic changes via the ACE2-Ang(1-7)-Mas axis. a-HSCs, activated hepatic stellate cells; LSECs, liver sinusoidal endothelial cells.

It has been demonstrated that neuroendocrine regulation plays a significant role in the process of liver fibrosis (Figure 2 and Figure 3). In this review, we illustrate the complex relationship between HSCs, cholangiocytes, myofibroblasts, ECM, and liver fibrosis. Quiescent state HSCs are activated and further converted into myofibroblasts in response to liver injury. Myofibroblasts are a key factor in fibrotic disease because myofibroblast cells produce ECM and form a fibrous scar at the site of injury. If the injury persists, excessive ECM accumulates and eventually leads to the formation of liver fibrosis. The fibrotic liver will return to a normal state when the etiological factors subside.

The role of neuroendocrine regulation has become an emerging concern in the treatment of various liver diseases, including liver fibrosis and cirrhosis, which may regulate the proliferation and apoptosis of hepatocytes or cholangiocytes and provide new approaches for the treatment of liver fibrosis. The discussion in this review illustrates that different neuropeptides exert different effects in the process of liver fibrosis, which may be related to different binding receptors, cell-cell communication, or different functions in distinct stages of the disease. The effects of many neuropeptides in liver fibrosis are still controversial. In addition, there are other neuropeptides that are abnormally expressed in the liver fibrosis process, but their function in liver disease is not yet understood. Further studies need to be performed before neuropeptides can be applied in the treatment of liver fibrosis.

In conclusion, the effect of neuroendocrine regulation on liver fibrosis provides a new prospect for the future clinical treatment of chronic liver diseases. In order to make important progress in the management of this kind of human disorder, a deeper understanding of these complex and relevant mechanisms is necessary to drive progress toward novel interventions.

## Data Availability

Data sharing is not applicable to this article as no datasets were generated or analyzed during the current study.

## Abbreviations

AANAT, *N*-acetyltransferase; ACE, angiotensin-converting enzyme; a-HSCs, activated hepatic stellate cells; Ang, angiotensin; AT1, angiotensin type 1 receptor; BDL, bile duct ligation; cAMP, cyclic adenosine 3′5′-monophosphate; CB, cannabinoids; CB1, cannabinoid receptor 1; CCL4, carbon tetrachloride; COX-2, cyclooxygenase-2; ECM, extracellular matrix; ECs, endocannabinoids; EMT, epithelial-mesenchymal transition; GalR, galanin receptor; HDC, histidine decarboxylase; HGF, hepatocyte growth factor; HR, Histamine receptor; HSCs, hepatic stellate cells; IBDM, intrahepatic bile duct mass; LSECs, liver sinusoidal endothelial cells; JNK, Junn-terminal protein kinase; MAPK, mitogen-activated protein kinase; Mdr2^−/−^, multidrug resistance gene 2 knockout; MMPs, matrix metalloproteinases; MT, melatonin receptor; NK-1R, neurokinin-1 receptor; NOX, NADPH oxidase; NO, nitric oxide; NPY, neuropeptide Y; Nrf2, nuclear factor erythroid 2-related factor 2; OR, opioid receptors; PBC, primary biliary cirrhosis; PDGF, platelet-derived growth factor; PKC, protein kinase C; PSC, primary sclerosing cholangitis; q-HSCs, quiet HSCs; RAS, renin-angiotensin system; RORα, retinoic acid receptor-related orphan receptor-α; ROS, reactive oxygen species; Sct, secretin; SERT, serotonin transporter; SP, substance P; SR, secretin receptor; TGF-β, transforming growth factor; TIMPs, tissue inhibitors of metalloproteinases; Y1, neuropeptide Y receptor 1; α-CGRP, α-calcitonin gene-related peptide; α-SMA, α-smooth muscle actin; 5-HT, 5-hydroxytryptamine; 5-HT_1,_ 5-hydroxytryptamine receptor families 1.

## References

[1] Racanelli V., Rehermann B. (2006). The liver as an immunological organ. Hepatology.

[2] Kisseleva T., Brenner D.A. (2008). Mechanisms of fibrogenesis. Exp. Biol. Med..

[3] Jensen K.J., Alpini G., Glaser S. (2013). Hepatic nervous system and neurobiology of the liver. Compr. Physiol..

[4] Stoyanova I.I., Gulubova M.V. (2000). Immunocytochemical study on the liver innervation in patients with cirrhosis. Acta Histochem..

[5] Iredale J.P. (2007). Models of liver fibrosis: Exploring the dynamic nature of inflammation and repair in a solid organ. J. Clin. Investig..

[6] Bataller R., Brenner D.A. (2005). Liver fibrosis. J. Clin. Investig..

[7] Parsonage G., Filer A.D., Haworth O., Nash G.B., Rainger G.E., Salmon M., Buckley C.D. (2005). A stromal address code defined by fibroblasts. Trends Immunol..

[8] Sempowski G.D., Borrello M.A., Blieden T.M., Barth R.K., Phipps R.P. (1995). Fibroblast heterogeneity in the healing wound. Wound Repair Regen..

[9] Kinnman N., Housset C. (2002). Peribiliary myofibroblasts in biliary type liver fibrosis. Front. Biosci..

[10] Friedman S.L. (2010). Evolving challenges in hepatic fibrosis. Nat. Rev. Gastroenterol. Hepatol..

[11] Iwaisako K., Jiang C., Zhang M., Cong M., Moore-Morris T.J., Park T.J., Liu X., Xu J., Wang P., Paik Y.H. (2014). Origin of myofibroblasts in the fibrotic liver in mice. Proc. Natl. Acad. Sci. USA.

[12] Zeisberg M., Yang C., Martino M., Duncan M.B., Rieder F., Tanjore H., Kalluri R. (2007). Fibroblasts derive from hepatocytes in liver fibrosis via epithelial to mesenchymal transition. J. Biol. Chem..

[13] Chu A.S., Diaz R., Hui J.J., Yanger K., Zong Y., Alpini G., Stanger B.Z., Wells R.G. (2011). Lineage tracing demonstrates no evidence of cholangiocyte epithelial-to-mesenchymal transition in murine models of hepatic fibrosis. Hepatology.

[14] Scholten D., Osterreicher C.H., Scholten A., Iwaisako K., Gu G., Brenner D.A., Kisseleva T. (2010). Genetic labeling does not detect epithelial-to-mesenchymal transition of cholangiocytes in liver fibrosis in mice. Gastroenterology.

[15] Benyon R.C., Iredale J.P. (2000). Is liver fibrosis reversible?. Gut.

[16] McCawley L.J., Matrisian L.M. (2001). Matrix metalloproteinases: They’re not just for matrix anymore!. Curr. Opin. Cell Biol..

[17] Hemmann S., Graf J., Roderfeld M., Roeb E. (2007). Expression of MMPs and TIMPs in liver fibrosis—A systematic review with special emphasis on anti-fibrotic strategies. J. Hepatol..

[18] Arthur M.J. (2000). Fibrogenesis II. Metalloproteinases and their inhibitors in liver fibrosis. Am. J. Physiol. Liver Physiol..

[19] Zhang C.Y., Yuan W.G., He P., Lei J.H., Wang C.X. (2016). Liver fibrosis and hepatic stellate cells: Etiology, pathological hallmarks and therapeutic targets. World J. Gastroenterol..

[20] Marra F. (1999). Hepatic stellate cells and the regulation of liver inflammation. J. Hepatol..

[21] Aydin M.M., Akcali K.C. (2018). Liver fibrosis. Turk. J. Gastroenterol..

[22] Maroni L., Haibo B., Ray D., Zhou T., Wan Y., Meng F., Marzioni M., Alpini G. (2015). Functional and structural features of cholangiocytes in health and disease. Cell. Mol. Gastroenterol. Hepatol..

[23] Alvaro D., Mancino M.G., Glaser S., Gaudio E., Marzioni M., Francis H., Alpini G. (2007). Proliferating cholangiocytes: A neuroendocrine compartment in the diseased liver. Gastroenterology.

[24] Krizhanovsky V., Yon M., Dickins R.A., Hearn S., Simon J., Miething C., Yee H., Zender L., Lowe S.W. (2008). Senescence of activated stellate cells limits liver fibrosis. Cell.

[25] Fallowfield J.A., Mizuno M., Kendall T.J., Constandinou C.M., Benyon R.C., Duffield J.S., Iredale J.P. (2007). Scar-associated macrophages are a major source of hepatic matrix metalloproteinase-13 and facilitate the resolution of murine hepatic fibrosis. J. Immunol..

[26] Roskams T., Cassiman D., De Vos R., Libbrecht L. (2004). Neuroregulation of the neuroendocrine compartment of the liver. Anat. Rec. A Discov. Mol. Cell Evol. Biol..

[27] Marzioni M., Fava G., Benedetti A. (2006). Nervous and Neuroendocrine regulation of the pathophysiology of cholestasis and of biliary carcinogenesis. World J. Gastroenterol..

[28] Micera A., Puxeddu I., Lambiase A., Antonelli A., Bonini S., Bonini S., Aloe L., Pe’Er J., Levi-Schaffer F. (2005). The pro-fibrogenic effect of nerve growth factor on conjunctival fibroblasts is mediated by transforming growth factor-beta. Clin. Exp. Allergy.

[29] Lee Y.A., Wallace M.C., Friedman S.L. (2015). Pathobiology of liver fibrosis: A translational success story. Gut.

[30] Kisseleva T., Brenner D. (2021). Molecular and cellular mechanisms of liver fibrosis and its regression. Nat. Rev. Gastroenterol. Hepatol..

[31] Geerts A. (2001). History, heterogeneity, developmental biology, and functions of quiescent hepatic stellate cells. Semin. Liver Dis..

[32] Cassiman D., van Pelt J., De Vos R., Van Lommel F., Desmet V., Yap S.H., Roskams T. (1999). Synaptophysin: A novel marker for human and rat hepatic stellate cells. Am. J. Pathol..

[33] Roskams T.A., Libbrecht L., Desmet V.J. (2003). Progenitor cells in diseased human liver. Semin. Liver Dis..

[34] Roskams T., De Vos R., van den Oord J.J., Desmet V. (1991). Cells with neuroendocrine features in regenerating human liver. APMIS Suppl..

[35] Hall J.E., Guyton A.C., Mizelle H.L. (1990). Role of the renin-angiotensin system in control of sodium excretion and arterial pressure. Acta Physiol. Scand. Suppl..

[36] Kaschina E., Unger T. (2003). Angiotensin AT1/AT2 receptors: Regulation, signalling and function. Blood Press..

[37] Santos R.A., Brosnihan K.B., Chappell M.C., Pesquero J., Chernicky C.L., Greene L.J., Ferrario C.M. (1988). Converting enzyme activity and angiotensin metabolism in the dog brainstem. Hypertension.

[38] Donoghue M., Hsieh F., Baronas E., Godbout K., Gosselin M., Stagliano N., Donovan M., Woolf B., Robison K., Jeyaseelan R. (2000). A novel angiotensin-converting enzyme-related carboxypeptidase (ACE2) converts angiotensin I to angiotensin 1-9. Circ. Res..

[39] Santos R.A., Haibara A.S., Campagnole-Santos M.J., Simoes e Silva A.C., Paula R.D., Pinheiro S.V., Leite M.F., Lemos V.S., Silva D.M., Guerra M.T. (2003). Characterization of a new selective antagonist for angiotensin-(1-7), D-pro7-angiotensin-(1-7). Hypertension.

[40] Pereira R.M., Dos Santos R.A., Teixeira M.M., Leite V.H., Costa L.P., da Costa Dias F.L., Barcelos L.S., Collares G.B., Simoes e Silva A.C. (2007). The renin-angiotensin system in a rat model of hepatic fibrosis: Evidence for a protective role of Angiotensin-(1-7). J. Hepatol..

[41] Simões E., Silva A.C., Miranda A.S., Rocha N.P., Teixeira A.L. (2017). Renin angiotensin system in liver diseases: Friend or foe?. World J. Gastroenterol..

[42] Leung P.S. (2004). The peptide hormone angiotensin II: Its new functions in tissues and organs. Curr. Protein Pept. Sci..

[43] Bataller R., Gines P., Nicolas J.M., Gorbig M.N., Garcia-Ramallo E., Gasull X., Bosch J., Arroyo V., Rodes J. (2000). Angiotensin II induces contraction and proliferation of human hepatic stellate cells. Gastroenterology.

[44] Tharaux P.L., Chatziantoniou C., Fakhouri F., Dussaule J.C. (2000). Angiotensin II activates collagen I gene through a mechanism involving the MAP/ER kinase pathway. Hypertension.

[45] Cai S.M., Yang R.Q., Li Y., Ning Z.W., Zhang L.L., Zhou G.S., Luo W., Li D.H., Chen Y., Pan M.X. (2016). Angiotensin-(1-7) Improves Liver Fibrosis by Regulating the NLRP3 Inflammasome via Redox Balance Modulation. Antioxid. Redox Signal..

[46] Centonze D., Finazzi-Agro A., Bernardi G., Maccarrone M. (2007). The endocannabinoid system in targeting inflammatory neurodegenerative diseases. Trends Pharmacol. Sci..

[47] Van Sickle M.D., Duncan M., Kingsley P.J., Mouihate A., Urbani P., Mackie K., Stella N., Makriyannis A., Piomelli D., Davison J.S. (2005). Identification and functional characterization of brainstem cannabinoid CB2 receptors. Science.

[48] Woods S.C. (2007). Role of the endocannabinoid system in regulating cardiovascular and metabolic risk factors. Am. J. Med..

[49] Mallat A., Lotersztajn S. (2006). Endocannabinoids as novel mediators of liver diseases. J. Endocrinol. Investig..

[50] Teixeira-Clerc F., Julien B., Grenard P., Tran V.N.J., Deveaux V., Li L., Serriere-Lanneau V., Ledent C., Mallat A., Lotersztajn S. (2006). CB1 cannabinoid receptor antagonism: A new strategy for the treatment of liver fibrosis. Nat. Med..

[51] Julien B., Grenard P., Teixeira-Clerc F., Van Nhieu J.T., Li L., Karsak M., Zimmer A., Mallat A., Lotersztajn S. (2005). Antifibrogenic role of the cannabinoid receptor CB2 in the liver. Gastroenterology.

[52] Siegmund S.V., Uchinami H., Osawa Y., Brenner D.A., Schwabe R.F. (2005). Anandamide induces necrosis in primary hepatic stellate cells. Hepatology.

[53] Acuna-Castroviejo D., Escames G., Venegas C., Diaz-Casado M.E., Lima-Cabello E., Lopez L.C., Rosales-Corral S., Tan D.X., Reiter R.J. (2014). Extrapineal melatonin: Sources, regulation, and potential functions. Cell. Mol. Life Sci..

[54] Renzi A., Glaser S., DeMorrow S., Mancinelli R., Meng F., Franchitto A., Venter J., White M., Francis H., Han Y. (2011). Melatonin inhibits cholangiocyte hyperplasia in cholestatic rats by interaction with MT1 but not MT2 melatonin receptors. Am. J. Physiol.-Gastrointest. Liver Physiol..

[55] Crespo I., San-Miguel B., Fernandez A., Ortiz de Urbina J., Gonzalez-Gallego J., Tunon M.J. (2015). Melatonin limits the expression of profibrogenic genes and ameliorates the progression of hepatic fibrosis in mice. Transl. Res..

[56] Shajari S., Laliena A., Heegsma J., Tunon M.J., Moshage H., Faber K.N. (2015). Melatonin suppresses activation of hepatic stellate cells through RORalpha-mediated inhibition of 5-lipoxygenase. J. Pineal Res..

[57] Kang J.W., Hong J.M., Lee S.M. (2016). Melatonin enhances mitophagy and mitochondrial biogenesis in rats with carbon tetrachloride-induced liver fibrosis. J. Pineal Res..

[58] Wu N., Meng F., Zhou T., Han Y., Kennedy L., Venter J., Francis H., DeMorrow S., Onori P., Invernizzi P. (2017). Prolonged darkness reduces liver fibrosis in a mouse model of primary sclerosing cholangitis by miR-200b down-regulation. FASEB J..

[59] Steinhoff M.S., von Mentzer B., Geppetti P., Pothoulakis C., Bunnett N.W. (2014). Tachykinins and their receptors: Contributions to physiological control and the mechanisms of disease. Physiol. Rev..

[60] Wan Y., Meng F., Wu N., Zhou T., Venter J., Francis H., Kennedy L., Glaser T., Bernuzzi F., Invernizzi P. (2017). Substance P increases liver fibrosis by differential changes in senescence of cholangiocytes and hepatic stellate cells. Hepatology.

[61] Glaser S., Gaudio E., Renzi A., Mancinelli R., Ueno Y., Venter J., White M., Kopriva S., Chiasson V., DeMorrow S. (2011). Knockout of the neurokinin-1 receptor reduces cholangiocyte proliferation in bile duct-ligated mice. Am. J. Physiol. Gastrointest. Liver Physiol..

[62] Peng L., Jia X., Zhao J., Cui R., Yan M. (2017). Substance P promotes hepatic stellate cell proliferation and activation via the TGF-beta1/Smad-3 signaling pathway. Toxicol. Appl. Pharmacol..

[63] Piao J., Jeong J., Jung J., Yoo K., Hong H.S. (2019). Substance P Promotes Liver Sinusoidal Endothelium-Mediated Hepatic Regeneration by NO/HGF Regulation. J. Interferon Cytokine Res..

[64] Hoyer D., Hannon J.P., Martin G.R. (2002). Molecular, pharmacological and functional diversity of 5-HT receptors. Pharmacol. Biochem. Behav..

[65] Lesurtel M., Graf R., Aleil B., Walther D.J., Tian Y., Jochum W., Gachet C., Bader M., Clavien P.A. (2006). Platelet-derived serotonin mediates liver regeneration. Science.

[66] Ruddell R.G., Oakley F., Hussain Z., Yeung I., Bryan-Lluka L.J., Ramm G.A., Mann D.A. (2006). A role for serotonin (5-HT) in hepatic stellate cell function and liver fibrosis. Am. J. Pathol..

[67] Pang Q., Jin H., Wang Y., Dai M., Liu S., Tan Y., Liu H., Lu Z. (2021). Depletion of serotonin relieves concanavalin A-induced liver fibrosis in mice by inhibiting inflammation, oxidative stress, and TGF-beta1/Smads signaling pathway. Toxicol. Lett..

[68] Polat B., Halici Z., Cadirci E., Karakus E., Bayir Y., Albayrak A., Unal D. (2017). Liver 5-HT7 receptors: A novel regulator target of fibrosis and inflammation-induced chronic liver injury in vivo and in vitro. Int. Immunopharmacol..

[69] Bracq S., Clement B., Pidoux E., Moukhtar M.S., Jullienne A. (1994). CGRP is expressed in primary cultures of human hepatocytes and in normal liver. FEBS Lett..

[70] Linscheid P., Seboek D., Zulewski H., Keller U., Muller B. (2005). Autocrine/paracrine role of inflammation-mediated calcitonin gene-related peptide and adrenomedullin expression in human adipose tissue. Endocrinology.

[71] Barry C.M., Kestell G., Gillan M., Haberberger R.V., Gibbins I.L. (2015). Sensory nerve fibers containing calcitonin gene-related peptide in gastrocnemius, latissimus dorsi and erector spinae muscles and thoracolumbar fascia in mice. Neuroscience.

[72] Goehler L.E., Sternini C. (1996). Calcitonin gene-related peptide innervation of the rat hepatobiliary system. Peptides.

[73] Bendtsen F., Schifter S., Henriksen J.H. (1991). Increased circulating calcitonin gene-related peptide (CGRP) in cirrhosis. J. Hepatol..

[74] Glaser S.S., Ueno Y., DeMorrow S., Chiasson V.L., Katki K.A., Venter J., Francis H.L., Dickerson I.M., DiPette D.J., Supowit S.C. (2007). Knockout of alpha-calcitonin gene-related peptide reduces cholangiocyte proliferation in bile duct ligated mice. Lab. Invest..

[75] Yoon S.P., Kim J. (2018). Exogenous CGRP upregulates profibrogenic growth factors through PKC/JNK signaling pathway in kidney proximal tubular cells. Cell Biol. Toxicol..

[76] Wan Y., Ceci L., Wu N., Zhou T., Chen L., Venter J., Francis H., Bernuzzi F., Invernizzi P., Kyritsi K. (2019). Knockout of α-calcitonin gene-related peptide attenuates cholestatic liver injury by differentially regulating cellular senescence of hepatic stellate cells and cholangiocytes. Lab. Investig..

[77] Inoue N., Magari S., Ito Y., Sakanaka M. (1989). Distribution, possible origins and fine structure of neuropeptide Y-containing nerve fibers in the rat liver. Brain Res..

[78] Wong P.F., Gall M.G., Bachovchin W.W., McCaughan G.W., Keane F.M., Gorrell M.D. (2016). Neuropeptide Y is a physiological substrate of fibroblast activation protein: Enzyme kinetics in blood plasma and expression of Y2R and Y5R in human liver cirrhosis and hepatocellular carcinoma. Peptides.

[79] Andrews Z.B., Liu Z.W., Walllingford N., Erion D.M., Borok E., Friedman J.M., Tschop M.H., Shanabrough M., Cline G., Shulman G.I. (2008). UCP2 mediates ghrelin’s action on NPY/AgRP neurons by lowering free radicals. Nature.

[80] Zheng F., Kim Y.J., Chao P.T., Bi S. (2013). Overexpression of neuropeptide Y in the dorsomedial hypothalamus causes hyperphagia and obesity in rats. Obesity.

[81] Mao Y., Zhang S., Yu F., Li H., Guo C., Fan X. (2015). Ghrelin Attenuates Liver Fibrosis through Regulation of TGF-beta1 Expression and Autophagy. Int. J. Mol. Sci..

[82] DeMorrow S., Onori P., Venter J., Invernizzi P., Frampton G., White M., Franchitto A., Kopriva S., Bernuzzi F., Francis H. (2011). Neuropeptide Y inhibits cholangiocarcinoma cell growth and invasion. Am. J. Physiol. Cell Physiol..

[83] DeMorrow S., Meng F., Venter J., Leyva-Illades D., Francis H., Frampton G., Pae H.Y., Quinn M., Onori P., Glaser S. (2013). Neuropeptide Y inhibits biliary hyperplasia of cholestatic rats by paracrine and autocrine mechanisms. Am. J. Physiol. Gastrointest. Liver Physiol..

[84] Dai W., Liu Y., Zhang Y., Sun Y., Sun C., Zhang Y., Lv X. (2019). Expression of neuropeptide Y is increased in an activated human HSC cell line. Sci. Rep..

[85] Smyth D.G. (2016). 60 YEARS OF POMC: Lipotropin and beta-endorphin: A perspective. J. Mol. Endocrinol..

[86] Vaccarino A.L., Kastin A.J. (2000). Endogenous opiates: 1999. Peptides.

[87] Mani A.R., Rasool R., Montagnese S., Dehpour A.R. (2006). Endogenous opioids and liver disease. Scand. J. Gastroenterol..

[88] Marzioni M., Svegliati B.G., Alpini G., Benedetti A. (2007). Endogenous opioid peptides and chronic liver disease: From bedside to bench. J. Hepatol..

[89] Marzioni M., Alpini G., Saccomanno S., de Minicis S., Glaser S., Francis H., Trozzi L., Venter J., Orlando F., Fava G. (2006). Endogenous opioids modulate the growth of the biliary tree in the course of cholestasis. Gastroenterology.

[90] Bergasa N.V., Rothman R.B., Vergalla J., Xu H., Swain M.G., Jones E.A. (1992). Central mu-opioid receptors are down-regulated in a rat model of cholestasis. J. Hepatol..

[91] Bergasa N.V., Sabol S.L., Young W.S., Kleiner D.E., Jones E.A. (1995). Cholestasis is associated with preproenkephalin mRNA expression in the adult rat liver. Am. J. Physiol..

[92] Bergasa N.V., Liau S., Homel P., Ghali V. (2002). Hepatic Met-enkephalin immunoreactivity is enhanced in primary biliary cirrhosis. Liver.

[93] De Minicis S., Candelaresi C., Marzioni M., Saccomano S., Roskams T., Casini A., Risaliti A., Salzano R., Cautero N., di Francesco F. (2008). Role of endogenous opioids in modulating HSC activity in vitro and liver fibrosis in vivo. Gut.

[94] Ebrahimkhani M.R., Kiani S., Oakley F., Kendall T., Shariftabrizi A., Tavangar S.M., Moezi L., Payabvash S., Karoon A., Hoseininik H. (2006). Naltrexone, an opioid receptor antagonist, attenuates liver fibrosis in bile duct ligated rats. Gut.

[95] Ch’Ng J.L., Christofides N.D., Anand P., Gibson S.J., Allen Y.S., Su H.C., Tatemoto K., Morrison J.F., Polak J.M., Bloom S.R. (1985). Distribution of galanin immunoreactivity in the central nervous system and the responses of galanin-containing neuronal pathways to injury. Neuroscience.

[96] Smith K.E., Walker M.W., Artymyshyn R., Bard J., Borowsky B., Tamm J.A., Yao W.J., Vaysse P.J., Branchek T.A., Gerald C. (1998). Cloned human and rat galanin GALR3 receptors. Pharmacology and activation of G-protein inwardly rectifying K+ channels. J. Biol. Chem..

[97] McMillin M., Frampton G., Grant S., DeMorrow S. (2017). The Neuropeptide Galanin Is Up-Regulated during Cholestasis and Contributes to Cholangiocyte Proliferation. Am. J. Pathol..

[98] Petrescu A.D., Grant S., Williams E., Frampton G., Parks N., Blaney H., Davies M., John R., Reinhart E.H., McMillin M. (2020). Coordinated Targeting of Galanin Receptors on Cholangiocytes and Hepatic Stellate Cells Ameliorates Liver Fibrosis in Multidrug Resistance Protein 2 Knockout Mice. Am. J. Pathol..

[99] He L., Li Z., Zhou D., Ding Y., Xu L., Chen Y., Fan J. (2016). Galanin receptor 2 mediates antifibrogenic effects of galanin on hepatic stellate cells. Exp. Ther. Med..

[100] Afroze S., Meng F., Jensen K., McDaniel K., Rahal K., Onori P., Gaudio E., Alpini G., Glaser S.S. (2013). The physiological roles of secretin and its receptor. Ann. Transl. Med..

[101] Glaser S., Lam I.P., Franchitto A., Gaudio E., Onori P., Chow B.K., Wise C., Kopriva S., Venter J., White M. (2010). Knockout of secretin receptor reduces large cholangiocyte hyperplasia in mice with extrahepatic cholestasis induced by bile duct ligation. Hepatology.

[102] Wu N., Meng F., Invernizzi P., Bernuzzi F., Venter J., Standeford H., Onori P., Marzioni M., Alvaro D., Franchitto A. (2016). The secretin/secretin receptor axis modulates liver fibrosis through changes in transforming growth factor-beta1 biliary secretion in mice. Hepatology.

[103] Wu N., Meng F., Zhou T., Venter J., Giang T.K., Kyritsi K., Wu C., Alvaro D., Onori P., Mancinelli R. (2018). The Secretin/Secretin Receptor Axis Modulates Ductular Reaction and Liver Fibrosis through Changes in Transforming Growth Factor-beta1-Mediated Biliary Senescence. Am. J. Pathol..

[104] Francis H., Meng F., Gaudio E., Alpini G. (2012). Histamine regulation of biliary proliferation. J. Hepatol..

[105] Francis H.L., Demorrow S., Franchitto A., Venter J.K., Mancinelli R.A., White M.A., Meng F., Ueno Y., Carpino G., Renzi A. (2012). Histamine stimulates the proliferation of small and large cholangiocytes by activation of both IP3/Ca^2+^ and cAMP-dependent signaling mechanisms. Lab. Investig..

[106] Jones H., Hargrove L., Kennedy L., Meng F., Graf-Eaton A., Owens J., Alpini G., Johnson C., Bernuzzi F., Demieville J. (2016). Inhibition of mast cell-secreted histamine decreases biliary proliferation and fibrosis in primary sclerosing cholangitis Mdr2^−/−^ mice. Hepatology.

[107] Kennedy L., Meadows V., Demieville J., Hargrove L., Virani S., Glaser S., Zhou T., Rinehart E., Jaeger V., Kyritsi K. (2020). Biliary damage and liver fibrosis are ameliorated in a novel mouse model lacking l-histidine decarboxylase/histamine signaling. Lab. Investig..

[108] de Lecea L., Criado J.R., Prospero-Garcia O., Gautvik K.M., Schweitzer P., Danielson P.E., Dunlop C.L., Siggins G.R., Henriksen S.J., Sutcliffe J.G. (1996). A cortical neuropeptide with neuronal depressant and sleep-modulating properties. Nature.

[109] Gonzalez-Rey E., Chorny A., Robledo G., Delgado M. (2006). Cortistatin, a new antiinflammatory peptide with therapeutic effect on lethal endotoxemia. J. Exp. Med..

[110] Wang M., Gong Q., Zhang J., Chen L., Zhang Z., Lu L., Yu D., Han Y., Zhang D., Chen P. (2017). Characterization of gene expression profiles in HBV-related liver fibrosis patients and identification of ITGBL1 as a key regulator of fibrogenesis. Sci. Rep..

[111] Benitez R., Caro M., Andres-Leon E., O’Valle F., Delgado M. (2022). Cortistatin regulates fibrosis and myofibroblast activation in experimental hepatotoxic- and cholestatic-induced liver injury. Br. J. Pharmacol..

